# Determination of Phenol with Peroxidase Immobilized on CaCO_3_

**DOI:** 10.3390/ijms24076766

**Published:** 2023-04-05

**Authors:** Aleksandr L. Kim, Alexey V. Dubrovskii, Egor V. Musin, Sergey A. Tikhonenko

**Affiliations:** Institute of Theoretical and Experimental Biophysics Russian Academy of Science, Institutskaya St., 3, 142290 Puschino, Moscow Region, Russia; kimerzent@gmail.com (A.L.K.); dav198@mail.ru (A.V.D.); eglork@gmail.com (E.V.M.)

**Keywords:** CaCO_3_ particles, enzyme detection system, peroxidase, phenol, phenol detection system

## Abstract

Phenols are widely used in industries despite their toxicity, which requires governments to limit their concentration in water to 5 mg/L before discharge to the city sewer. Thus, it is essential to develop a rapid, simple, and low-cost detection method for phenol. This study explored two pathways of peroxidase immobilization to develop a phenol detection system: peroxidase encapsulation into polyelectrolyte microcapsules and peroxidase captured by CaCO_3_. The encapsulation of peroxidase decreased enzyme activity by 96%; thus, this method cannot be used for detection systems. The capturing process of peroxidase by CaCO_3_ microspherulites did not affect the maximum reaction rate and the Michaelis constant of peroxidase. The native peroxidase—Vmax = 109 µM/min, Km = 994 µM; CaCO_3_–peroxidase—Vmax = 93.5 µM/min, Km = 956 µM. Ultimately, a reusable phenol detection system based on CaCO_3_ microparticles with immobilized peroxidase was developed, capable of detecting phenol in the range of 700 ng/mL to 14 µg/mL, with an error not exceeding 5%, and having a relatively low cost and production time. The efficiency of the system was confirmed by determining the content of phenol in a paintwork product.

## 1. Introduction

Anthropogenic activity has led to pollution in surface water [1] with metal ions, organic compounds, and other substances. This pollution has a negative impact on the ecosystem and human health [2]. One of the primary sources of pollution is industrial wastewater [3]. Therefore, it is essential to monitor the concentration of pollutants in industrial wastewater before it is released into the environment.

Phenol is one of the pollutants produced as a result of industrial activities. It is a by-product of various industries, including oil refining, gas and coke industries, pharmaceuticals, explosives, phenol–formaldehyde resins, plastics, and varnishes [4]. Phenol entering aquatic ecosystems adversely affects both aquatic organisms, including algae, protozoa, invertebrates and vertebrates, and humans [5]. Moderate concentrations (about 1 µg/mL) of phenol pollution may cause various diseases in humans, such as neurological, cardiac, respiratory, renal, and digestive diseases [6]. In addition, the presence of phenol may accelerate the growth of tumors. It should be noted that high concentrations of phenol can be fatal to humans (between 3 to 30 g) [7].

Due to the negative effects of phenol on the ecosystem and human health, laws in most countries limit its concentration in drinking water. Specifically, the upper limit for total phenol in drinking water is 0.5 µg/L (EU directive 80/778). For bathing water, the limit is 50 µg/L (directive 76/160/CEE), and for surface water intended for drinking, the limit is 1–100 µg/L (directive 75/440/CEE). Additionally, the concentration of phenol in industrial wastewater is also regulated to reduce its impact on the ecosystem. The allowable limit for phenol is 1 mg/L for industrial effluent discharges into inland surface waters (Indian standards (IS): 2490-1974) and 5 mg/L for municipal sewer discharges (IS: 3306-1974) [8].

Numerous studies of industrial waste have shown the presence of phenol in wastewater, with concentrations ranging from 50 to 2000 mg/L, well above the allowable concentration [9,10]. This indicates an incorrect wastewater treatment strategy. Therefore, it is necessary to determine the concentration of phenol in industrial effluents before they are discharged. The determination of phenol in water requires a fast, accurate, and sensitive procedure. The reference method is based on an oxidation reaction with 4-aminoantipyrine (4-AAP) and measuring absorbance at 510 nm [11]. This method has a limit of detection of 0.5 µg/L with extraction with chloroform and 1 µg/L with a direct photometric method. However, it cannot determine para-substituted phenols, and it requires the preliminary distillation of the sample to separate interfering substances. Additionally, it requires additional extraction using chloroform, which significantly slows down the analysis.

Several liquid or gas chromatography methods [12,13,14,15,16,17] offer high selectivity and low detection limits. However, they require multi-stage sampling, which results in longer analysis times. On the other hand, biosensors based on microorganisms [18], and immobilized enzymes [19,20] overcome this limitation. They enable quick analysis and reusable biosensors, but they either have insufficient sensitivity or are difficult to manufacture and maintain.

There are many studies dedicated to developing a phenol detection system, each of which tries to address the shortcomings described above in different ways. Some studies have focused on developing a detection system based on immobilized peroxidase. For example, Aybastıer, Ö. et al. suggested using immobilized peroxidase in chitosan beads [21]. This approach allows for the peroxidase-chitosan beads to be reused 10 times without a decrease in activity. However, this technology also has a long manufacturing time and a low sensitivity (1500 ng/mL). At the same time, Lindgren, A. et al. developed a peroxidase-modified graphite electrode that is free from some of these disadvantages [22]. The minimum detection concentration of phenol is 400 ng/mL, and the number of reuses reaches 30 times. However, this detection system has a long manufacturing time too. Sometimes, however, the manufacturing time is not important if the detection system has a high reuse number. For example, Yang, S. et al. developed an amperometric biosensor with immobilized peroxidase that was fixed by polyelectrolyte layers PSS/PAH/PSS/HRP [23]. This system may be used more than 900 times without significant loss of peroxidase activity. As a result, such a detection system significantly reduces the cost of analysis, but it has a low sensitivity level (2700 ng/mL). Therefore, it is still necessary to develop a new phenol detection system based on the immobilized peroxidase that does not have the disadvantages described above.

To monitor the concentration of phenol in industrial wastewater, it is necessary to develop new methods for its detection that are simple, fast, and inexpensive. The main aim of our work is to create a detection system based on encapsulated peroxidase into polyelectrolyte microcapsules (PMC) and captured peroxidase by CaCO_3_ microspherulite through the coprecipitation method [24].

## 2. Results and Discussions

**Figure 1:**
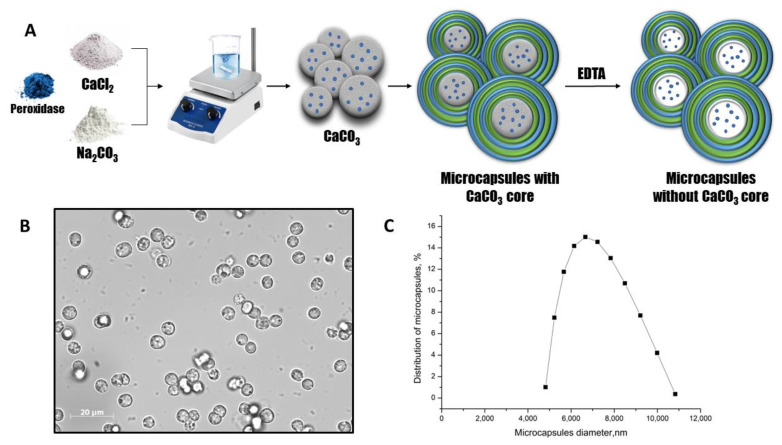
The scheme of the peroxidase encapsulation in polyelectrolyte microcapsules (**A**). The optical microscopy images of PMC (**B**). The PMC diameter distribution function (**C**).

Peroxidase was encapsulated in polyelectrolyte microcapsules using a layer-by-layer adsorption technique. Polystyrene sulfonate (PSS) and polyallylamine (PAH) were adsorbed onto a CaCO_3_ particle containing peroxidase, followed by the dissolution of the CaCO_3_ particle. Figure 1A shows the main scheme of the process for preparing polyelectrolyte microcapsules containing peroxidase.

The optical microscopy images of PMC (Figure 1B) demonstrate the morphological homogeneity of microcapsules and the absence of the CaCO_3_ core. The microcapsules containing peroxidase had an average diameter of 6.6 μm with a 25.1% polydispersity index (Figure 1C) and a ζ-potential of +27 ± 1 mV.

**Figure 2:**
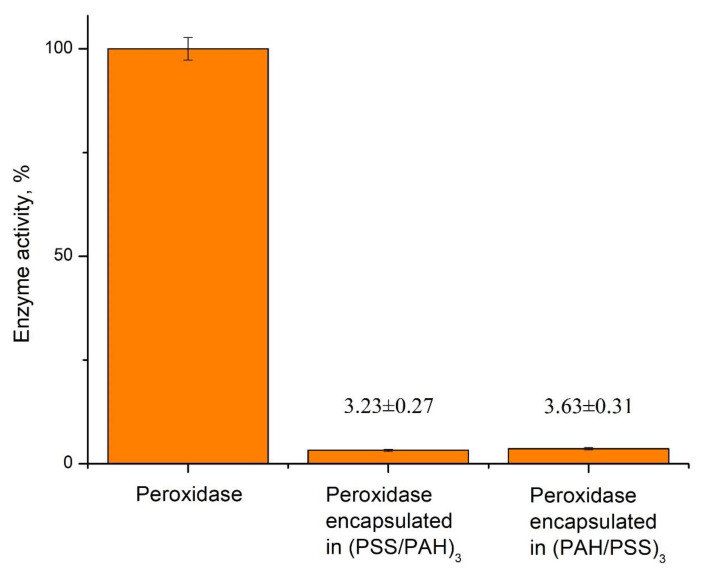
Activity of native and encapsulated peroxidase with different shell composition.

The microcapsules obtained had a composition of either (PSS/PAH)_3_ or (PAH/PSS)_3_, where PSS was the inner layer and PAH was the outer layer in (PSS/PAH)_3_, and vice versa for (PAH/PSS)_3_. The different inner and outer polyelectrolyte layers of PMC are necessary to study the influence of polyelectrolytes PSS and PAH on the encapsulated peroxidase activity. The first and last polyelectrolyte layers may have different extents of influence on the encapsulated enzyme, as demonstrated in the article of Sukhorukov B. I. et al. regarding encapsulation of urease and lactate dehydrogenase into polyelectrolyte microcapsules [25]. The activity of peroxidase encapsulated in PMCs with compositions of (PSS/PAH)_3_ and (PAH/PSS)_3_ was compared to that of native peroxidase. The results are presented in Figure 2.

As shown in Figure 2, the activity of the encapsulated enzyme significantly decreased compared to the native one. Additionally, the order of polyelectrolyte layers of PMC’s shell has affected peroxidase equally. Because of the low activity of the encapsulated enzyme, further development of the phenol detection system based on encapsulated peroxidase is not appropriate. 

**Figure 3:**
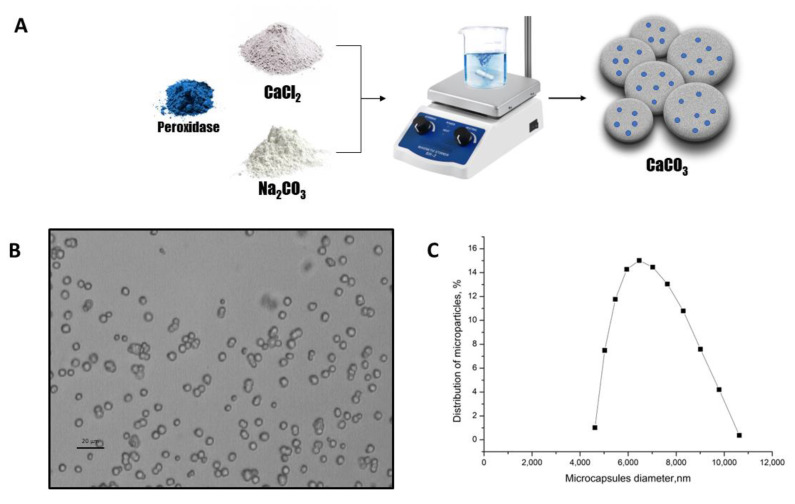
The scheme of the peroxidase-CaCO_3_ co-precipitation (**A**). The optical microscopy images of the CaCO_3_ microspherulites (**B**). The CaCO_3_ microspherulites diameter distribution function (**C**).

In connection with this, CaCO_3_ microparticles were proposed as carriers of peroxidase in this study. The peroxidase was captured using the co-precipitation method with CaCO_3_ microspherulites. This method involves co-precipitating the enzyme molecules with CaCO_3_ particles produced by mixing the Na_2_CO_3_ and CaCl_2_ solutions. Figure 3A shows the main scheme for preparing CaCO_3_ microspherulites containing peroxidase.

The optical microscopy images of the CaCO_3_ microspherulites (Figure 3B) demonstrate their morphological homogeneity. The average diameter of microspherulites containing peroxidase was 6.4 μm, with a 25.2% polydispersity index (Figure 3C), and their ζ-potential was +14 ± 1 mV.

**Figure 4:**
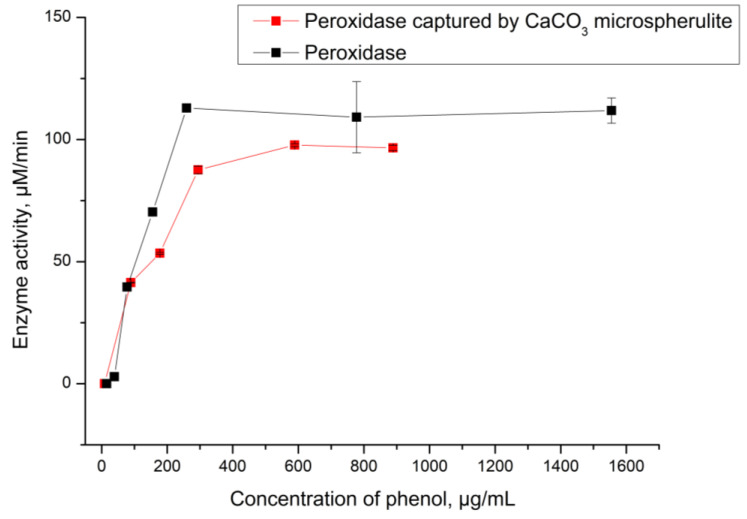
The activity of peroxidase captured by CaCO_3_ depending on phenol concentration.

Michaelis–Menten kinetics is one of the most well-known models of enzyme kinetics. It explains enzyme activity in relation to substrate concentration and enables the quantification of parameters that characterize enzyme efficiency. Two such parameters are Vmax, the maximum rate of enzyme reaction, and Km, which characterizes the enzyme’s affinity to the substrate [26]. In this study, we examined the Vmax and Km of native peroxidase and peroxidase captured in CaCO_3_ microspherulites. This allowed us to identify differences in kinetic parameters between native peroxidase and CaCO_3_–peroxidase, and to determine the suitability of CaCO_3_–peroxidase as a sensitive biological element in a detection system. To accomplish this, we measured peroxidase activity in the reaction of phenol oxidation at varying phenol concentrations (see Figure 4). In this reaction, peroxidase oxidizes phenol in the presence of hydrogen peroxide and 4-aminoantipyran, converting it into a red quinoneimine chromogen that changes the optical density of the solution [27].

As shown in Figure 4, the activity of native peroxidase and CaCO_3_–peroxidase is almost identical in both cases. The Michaelis constants are 94 µg/mL (equivalent to 994 µM) for the native enzyme and 90 µg/mL (equivalent to 956 µM) for the CaCO_3_–peroxidase. The lack of difference between these values indicates that the process of capturing peroxidase by CaCO_3_ microspherulites has not affected enzyme catalytic function. Therefore, the CaCO_3_–peroxidase can be used for further development of the phenol detection system.

**Figure 5:**
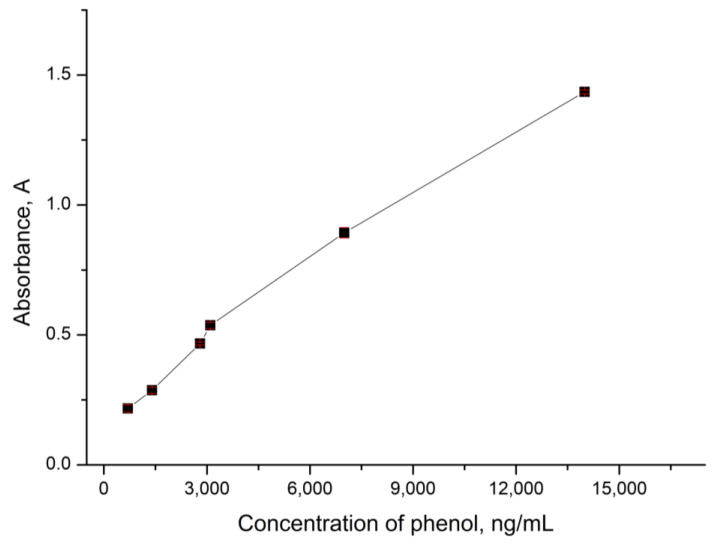
The optical density of the analyzed solution depending on the concentration of phenol.

In the next stage, we used the peroxidase captured by CaCO_3_ microspherulites to determine the concentration of phenol in the solution. To do this, CaCO_3_–peroxidase particles were incubated for 10 min in a solution containing phenol, hydrogen peroxide, and 4-aminoantipyran. After incubation, the CaCO_3_–peroxidase particles were precipitated by centrifugation and the optical density of the supernatant was determined. This allowed for us to detect the linear range of phenol concentrations, which is a necessary characteristic of a detection system as it enables us to understand the system’s detection limits. The results of this study are presented in Figure 5. 

Figure 5 shows that the optical density increases linearly with an increase in phenol concentration in the range from 700 ng/mL to 14 μg/mL. The approximating equation for the linear range of detectable phenol concentrations is y = 9 × 10^−5^x + 0.2001, with a standard deviation not exceeding 2% and approximation reliability coefficient of R² = 0.9888. This fact allows for us to conclude that CaCO_3_ microspherulites containing peroxidase can be used to analyze the phenol content in water in the concentration range from 700 ng/mL to 14 μg/mL.

**Figure 6:**
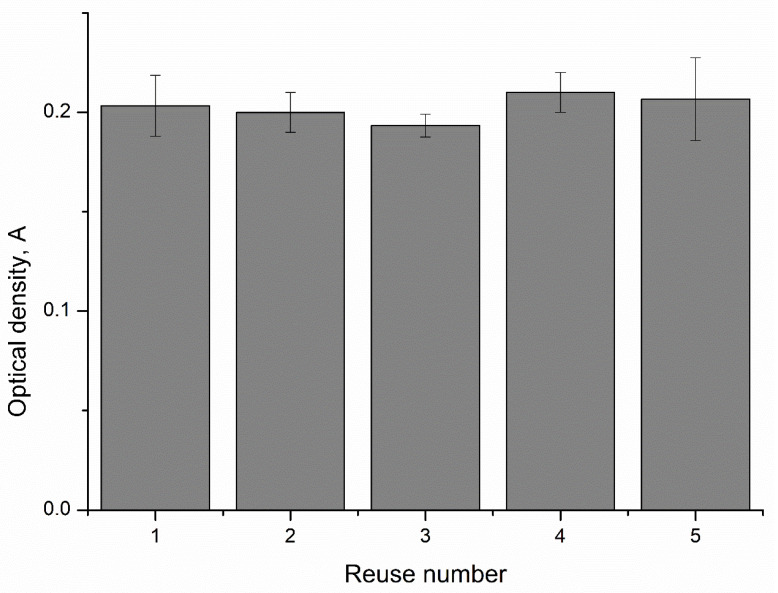
The reuse ability of CaCO_3_–peroxidase microparticles for the determination of phenol concentration.

One advantage of detection systems is their potential for reusable application. This advantage has been shown in detection systems based on encapsulated enzymes sensor [28,29,30,31,32,33,34], which reduce analysis costs by reducing enzyme consumption. To determine the feasibility of reusing CaCO_3_–peroxidase particles for phenol concentration detection, we incubated the same CaCO_3_–peroxidase particles five times in different solutions containing 700 ng/mL of phenol, and measured the optical density of each solution. The results of this study are presented in Figure 6.

As shown in Figure 6, the values of the optical densities after the reuse of CaCO_3_–peroxidase particles differ from each other by no more than 5%. This suggests that CaCO_3_ microspherulites containing peroxidase can be reused to determine the concentration of phenol in a solution.

In the final stage of our study, we investigated the possibility of using CaCO_3_–peroxidase to measure the phenol concentration in a phenol-contained liquid. For that purpose, we incubated CaCO_3_–peroxidase microparticles in a paint solution that was mixed with hydrogen peroxide and 4-aminoantipyran, and the optical density of the solution was determined. Additionally, the phenol concentration was measured with native peroxidase. In both cases, the optical density values coincided and amounted to 0.54 ± 0.021 A. Therefore, we can conclude that microspherulites containing peroxidase can be used to determine the concentration of phenol in phenol-containing liquids.

Thus, a detection system has been developed that can detect phenol concentrations ranging from 700 ng/mL to 14 µg/mL. This range is sufficient for determining phenol in industries such as oil refining, gas, and coke, as well as wastewater from industrial enterprises. In addition, CaCO_3_–peroxidase microparticles of the detection system can be reused, and the analysis time for one measurement is less than 10 minutes. The compared characteristics of our detection system are summarized in Table 1.

Table 1 shows that the CaCO_3_–peroxidase detection system has the lowest cost and shortest manufacturing time compared to other detection systems based on immobilized peroxidase. This makes it easy to scale up manufacturing processes for large-scale production. Although the CaCO_3_–peroxidase is 43% less sensitive than the most sensitive multiple-use detection system (no. 3), it can still detect phenol concentrations as low as 700 ng/mL. This makes it suitable for determining phenol in industrial wastewater, which is sufficient for most applications. However, CaCO_3_–peroxidase particles have a significant disadvantage: they cannot be used in low-pH solutions, as this will destroy the CaCO_3_ and release peroxidase into the solution. Therefore, it is necessary to use a buffer solution with a pH higher than 7.

## 3. Materials and Methods

### 3.1. Materials

The polyelectrolytes polystyrene sulfonate sodium (PSS) and polyallylamine (PAH) with a molecular mass of 70 kDa were purchased from Sigma (St. Louis, MO, USA). The horseradish peroxidase RZ 1,0 110 U/mg with a molecular weight 40 kDa was purchased from DIA-M (Moscow, MR, Russia). Sodium carbonate, phenol, aminoantipyrine-4, phosphate-buffered saline, hydrogen peroxide, and calcium chloride were obtained from “Reahim” (Moscow, MR, Russia).

### 3.2. Preparation of CaCO_3_ Microspherulites

A stirring of 0.33 M Na_2_CO_3_ was initiated, followed by the addition of 0.33 M of CaCl_2_ and 6 mg/mL of peroxidase. The mixture was stirred for 30 seconds and left to settle until the particles had completely precipitated [38]. The shape of the particles was checked using a light microscope to control the "ripening" process of the microspherolites. Then, the supernatant was decanted, and the precipitate was washed with water. The microparticles were obtained with an average diameter of 6.4 ± 2 μm. The number of microparticles and ζ-potential were measured using the dynamic light-scattering method on a Zetasizer nano ZS device (Malvern, United Kingdom). The size of microparticles was measured using the Light-Scattering Instrument for Particle Analysis Litesizer™ 500 (Anton Paar GmbH, Graz, Austria).

### 3.3. Preparation of Polyelectrolyte Microcapsules

The polyelectrolyte microcapsules (PMC) were obtained through a layer-by-layer process by adsorbing negatively or positively charged polyelectrolytes onto CaCO_3_ microspherulites, followed by the dissolution of CaCO_3_. The layer-by-layer adsorption of PAH and PSS on the CaCO_3_ microspherulites surface was carried out in polyelectrolytes solutions (concentration 2 mg/mL + 0.5 M NaCl). After each adsorption, the CaCO_3_ particles with adsorbed polyelectrolytes were triple-washed with a 0.5 M NaCl solution, which was necessary to remove unadsorbed polymer molecules. The particles were separated from the supernatant by centrifugation. After applying the required number of layers, the carbonate kernels were dissolved in a 0.2 M EDTA solution for 12 h. The resulting capsules were washed three times with water to remove core decay products. The microcapsules were obtained with an average diameter of 6.6 ± 2 μm. The number of microcapsules and ζ-potential were measured using the dynamic light-scattering method on a Zetasizer nano ZS device (Malvern, UK). The size of microcapsules was measured using the Light-Scattering Instrument for Particle Analysis Litesizer™ 500 (Anton Paar GmbH, Graz, Austria).

### 3.4. Measurement of Peroxidase Activity

Phenol of the required concentration was added to a solution of 0.1 M phosphate-buffered saline (pH 7.2) containing aminoantipyrine-4 0.3 mmol/L, hydrogen peroxide 4.68 mmol/L, and peroxidase 1.4 μg/mL (or 2.7 × 10^6^ particles of CaCO_3_ microspherulites with immobilized peroxidase, it is equal to 2.9 μg/mL of enzyme) at 24 °C to determine the activity of the enzyme [39]. This led to a change in optical density proportional to the concentration of phenol. The change in optical density at 510 nm was measured spectrophotometrically on a Cary 100 spectrophotometer for the first 20 s of the enzyme reaction.

### 3.5. Determination of Linear Range of Detectable Concentrations 

Phenol of the required concentration was added in a solution of 0.1 M phosphate-buffered saline (pH 7.2) containing aminoantipyrine-4 0.3 mmol/L, hydrogen peroxide 4.68 mmol/L and 2.7 × 10^6^ particles of CaCO_3_ microspherulites with immobilized peroxidase at 24 °C. It was incubated on a shaker for 10 min to determine the linear range of determined phenol concentrations using peroxidase captured by CaCO_3_ spherulites. After incubation, a suspension of microcapsules was precipitated by centrifugation (10,000× *g*, 1 min) and the optical density of the solution was measured at 510 nm on a Cary 100 spectrophotometer for the first 20 s of the enzyme reaction. 

For the measurement of phenol concentration in the “phenol-paint”, the phenol of the known concentration was changed to phenol paint 1.5%, which was diluted 5000 times and added in a solution of 0.1 M phosphate-buffered saline. All other steps are identical, as described above. Additionally, the native enzyme was used instead of the captured peroxidase by the CaCO_3_ spherulite as a control comparison.

### 3.6. Reuse of Peroxidase Captured by CaCO_3_ Microspherulites

Phenol (concentration 700 ng/mL) was added in a solution of 0.1 M phosphate-buffered saline (pH 7.2) containing aminoantipyrine-4 0.3 mmol/L, hydrogen peroxide 4.68 mmol/L, and 2.7 × 10^6^ particles of CaCO_3_ microspherulites with immobilized peroxidase and incubated on a shaker for 10 min to study the possibility of the repeated use of immobilized peroxidase. After incubation, a suspension of microcapsules was precipitated by centrifugation (10,000× *g* 1 min) and the optical density of the solution was measured at 510 nm on a Cary 100 spectrophotometer. However, at the same time, after the reaction, precipitation of spherulites and decontamination of the supernatant, a new analyzed solution with the same concentration of phenol, phosphate-buffered saline, aminoantipyrine, and hydrogen per-oxide was added to the sediment of spherulites. This procedure was repeated 5 times.

## 4. Conclusions

Detection systems for phenol concentration measuring are widely used in industries, but they have several issues: long manufacturing time, long and labor-intensive procedure of measuring, low sensitivity, and extra maintenance. Therefore, developing a new detection system that eliminates these drawbacks is necessary. In the present study, we researched the kinetic characteristics of peroxidase encapsulated in polyelectrolyte microcapsules and peroxidase captured by CaCO_3_ microspherulites, and developed a detection system for measuring the phenol concentration. 

In the first stage, we discovered that the peroxidase encapsulated in polyelectrolyte microcapsules (PSS/PAH)_3_ and (PAH/PSS)_3_ loses over 97% of its activity. Therefore, we concluded that the peroxidase encapsulated in polyelectrolyte microcapsules cannot be used for the development of detection systems. As a result, we proposed to develop the phenol detection system based on the peroxidase captured by CaCO_3_ microspherulites.

In the course of the work, we examined the kinetic characteristics of peroxidase captured by CaCO_3_ microspherulites (CaCO_3_–peroxidase) and compared them to those of native peroxidase. It was found that the maximum reaction velocity and the Michaelis–Menten constant differ insignificantly between native peroxidase and CaCO_3_–peroxidase: native peroxidase − Vmax = 1.14 (109.05 µM/min), Km = 94 (994 µM); CaCO_3_–peroxidase − Vmax = 1.03 (93.54 µM/min), Km = 90 (956 µM).

In the next stage, we used the peroxidase captured by CaCO_3_ microspherulites to determine the concentration of phenol in the solution, and determined the linear range of the determined phenol concentrations. It was found that the linear range is from 700 ng/mL to 14 μg/mL, with a standard deviation not exceeding 2% and the approximation reliability coefficient is R² = 0.9888. Additionally, it has been shown that the CaCO_3_ microspherulites containing peroxidase may be reused to determine the concentration of phenol in the solution with the measurement error of no more than 5%.

The developed detection system was tested by determining the concentration of phenol in the paint. The result of the study completely coincided with the results of the standard method using a native enzyme. Thus, the CaCO_3_–peroxidase detection system has the lowest time and cost of manufacturing compared to other phenol detection systems. Additionally, it allows for us to detect the phenol concentration from 700 ng/mL, which is sufficient for determining phenol in wastewater from industrial enterprises.

## Figures and Tables

**Table 1:** Compared characteristics of detection systems based on immobilized peroxidase.

№	Type of Peroxidase Biosensor	Activity Relatively to Native Peroxidase Activity, %	Km Relatively to Native Peroxidase Km, %	Minimal Concentration of Linearity, ng/mL	Reuse Number	Manufacturing Cost of Biosensor, $	Time of Manufacturing	Cost of 1 Analysis ***, $	References
1	Native Peroxidase	100	100	500	0	0.59	5 min	0.59	[35]
2	Chitosan beads with peroxidase	81	Lack of data	1500	10	4.87	27 h	0.487	[21]
3	Colorimetric Paper	Lack of data	Lack of data	1	0	52.95	6.5 h	52.95	[36]
4	Peroxidase-modified graphite electrode	Lack of data	47	400	30	3.63	32 h	0.121	[22]
5	SiO_2_/Nb_2_O_5_ sol-gel	Lack of data	Lack of data	470	200	53.17	6 h	0.26585	[37]
6	Amperometric biosensor PSS/PAH/PSS/HRP	Lack of data	Lack of data	2700	960 *	3.03	12 h	0.0031	[23]
7	CaCO3–peroxidase	100	100	700	10 **	0.13	20 min	0.013	Current article

* The reuse number calculated for 2 weeks of application (when detection system loses 24% of response) for 40 h work week. ** The reuse number limited by experiment. *** the cost of 1 analysis, taking into account the maximum reuse.

## Data Availability

Not applicable.
